# Acceptance of the use of silver fluoride among Brazilian parents of children with special health care needs

**DOI:** 10.3389/froh.2024.1377949

**Published:** 2024-05-30

**Authors:** N. Potgieter, V. Pereira, R. Elias, S. Charone, S. Groisman

**Affiliations:** ^1^ Department of Paediatric Dentistry, Faculty of Dentistry, University of the Western Cape, Cape Town, South Africa; ^2^ Dental Special Needs, Brazilian Dental Association of Duke de Caxias - Rio de Janeiro, Rio de Janeiro, Brazil; ^3^ Dental Special Needs, Brazilian Society of Duque de Caxias - Rio de Janeiro, Rio de Janeiro, Brazil; ^4^ Department of Community Dentistry, School of Dentistry, Federal University of Rio de Janeiro, Rio de Janeiro, Brazil; ^5^ DNA Laboratory of Diagnosis, Biosciences, Biology Institute, State University of Rio de Janeiro, Rio de Janeiro, Brazil

**Keywords:** silver diamine fluoride (SDF), silver fluoride, parental acceptance, children with special health care needs (CSHCN), caries arrest

## Abstract

**Background:**

Children with special health care needs including Down Syndrome, Autism Spectrum Disorder and Down Syndrome experience difficulties in receiving dental treatment. Silver Diamine Fluoride (SDF) and Silver Fluoride (SF) are a minimally invasive treatments options to arrest dental caries without sedation; local or general anaesthesia (GA).

**Aim:**

Evaluation of Brazilian's parents' acceptance of the use of SF in CSHCN.

**Methods:**

After receiving education on SF, 100 Parents of CSHCN completed a questionnaire concerning their acceptance of SF, in different dental situation.

**Result:**

Majority of parents (74,5%) agreed to the use of SF for their children. SF was more acceptable on posterior teeth (74,5%) when compared to its use on anterior teeth (43,1%). Parents accepted to use SF in order: to reduce infection and pain (82,4%); to avoid dental injection (72,5%) and treatment under GA (84,3%). The Majority of parents accepted the properties of SF (82,4%) and Silver (80,4%).

**Conclusion:**

Silver Fluoride was accepted as a treatment option for caries, by Brazilian parents of CSHCN. SF should be considered as a treatment option for caries limited to dentine for CSHCN, taking into consideration the individual needs and opinions with regard to aesthetics and exposure to fluoride and silver.

## 1 Introduction

According World Health Organization (WHO), approximately 15% of global population lives with disabilities or special needs, indicating more than 1 billion people (1).

The Centers for Disease Control and Prevention, described that 1 in 68 children suffer from autism spectrum disorder (ASD) in the United States (2). The increase in prevalence could be due to better diagnostic criteria, as well genetic diagnosis (3, 4). Several studies have highlighted the difficulties of performing dental care on children with a serious mental disability like Cerebral Paralysis (CP), Down Syndrome (DS) and Autism Spectrum Disorder (ASD) (2, 4–6). CSHCN, tends to receive less oral health care than general population, which can lead to a poorer oral health outcomes, impacting nutrition, general diseases and quality of life (7).

Delivering good oral care to CSHCN has many challenges, not only concerning their medical status, but also the family's involvement in attaining proper oral care, transportation difficulties among others (8). The most common reason cited for not taking children to the dentist for DS group was “Not aware of the dental problems of their children” and for non- DS groups “No awareness of the importance of dental visit” (61.2% and 53%, respectively) (8). It has also being reported that individuals with special needs demonstrate exaggerated responses to physical stimulation, especially with regards to oral sensitivity (9, 10). This hypersensitivity to oral stimulation poses a challenge for dentists in providing proper dental treatment.

Silver Diamine Fluoride (SDF), is a product that has gained popularity as a minimally invasive treatment option and has demonstrated effectiveness in arresting dental caries in clinical trials (11). The product was originally used for desensitization but has proven effectiveness in arresting dental caries (11). SDF is also cost effective, requires limited instruments and does not require anaesthesia or cause severe pain during application (11).

The new generation ammonia free Silver Fluoride (SF) has added benefits, including less risk for tissue burn and irritation, improved smell, and improved product stability (12). Both Silver Diamine Fluoride (Riva Star, SDI Limited, Australia) and Silver Fluoride (Riva Star Aqua, SDI Limited, Australia) is approved for medical use with product registration numbers (D349082/K172047/GMDN code 45232 Class iia), and the qualities are summarized in Table 1 (12).

**Table 1:** A comparative summary of riva star and riva star aqua.

	Riva star (SDF)	Riva star aqua (SF)
Formulation	38% Silver Fluoride in an ammonia solution; 5% Fluoride	38% Silver Fluoride in water; 5% Fluoride
pH levels	High (+-pH = 10)	Physiological (+-pH = 7)
Storage requirements	Refrigerated	Room temperature
Odour/Taste	Ammonia taste and odour	No ammonia taste and odour
Tissue irritation	Mild	None

The improved properties of SF makes it even more ideal for CSHCN as the oral stimulation is less than SDF with regards to taste, smell and the “burning” sensation. Limited research is available on the acceptancy of parents or caregivers, concerning the use of SF as well as the efficacy of the treatment in CSHCN. The aim of this study was to identify whether there is parental acceptance of SF as a treatment option for CSHCN in Rio de Janeiro, Brazil. A subsequent study then evaluated the clinical success of SF application to carious lesions among this vulnerable group of our population.

Duque de Caxias, is a Brazilian Municipality in the State of Rio de Janeiro, southeast Region of the country. It is located in Baixada Fluminense, in the Metropolitan Region of Rio de Janeiro, 16 km from the state capital. Its population in 2023 according to National Census is 808,152 inhabitants, making it the most populous in the state and 22nd most populous in the country (13). In Duke de Caxias_,_ 18,07% of individual received 2,6 minimum salary and 37.8% of the population recives half minimum salary per month 2010 (14). The minimum Salary is the lowest national wage mandatory by Brazilian by Law, and one minumum salary in Brazil is 272,30 Euros (15).

## 2 Materials and methods

The aim of this *in vivo* study was to determine the acceptance of the parents/legal guardians of SF application as treatment option for Brazilian CSHCN.

Ethical approval was obtained by the Ethical Committee Approval (CEP-Dental Association of Duque de Caxias).

This cross-sectional, questionnaire based study included 100 participants. All the participants self-reported to the Association of Dentistry: Duque de Caxias-Rio de Janeiro, Brazil for dental treatment. This association is dedicated to provide treatment to any patient, including patients without medical insurance. The study was conducted over a period 6 months from January 8th 2022–June 7th 2022.

Parents of children diagnosed with DS, CP and ASD, that required dental treatment, with at least one cavity indicated for restoration, were invited to participate in the study. All participants were informed about the purpose and nature of the study. All participants where from the same municipality with the same socio-economic status, therefore no statistical test was performed to observed if might be same variable that could affected the results of the acceptance of SF.

Once written informed consent was obtained for participation, the parents were educated about the treatment option of SF by making use of a patient information leaflet adapted from the British Society of Paediatric Dentistry's (16). The questionnaire was printed in English but a translator was available when necessary. The questionnaire was adapted from Crystal et al. (17), and evaluated the general acceptance of SF as a treatment option for caries, aesthetic concerns and concerns related to the composition of the SF treatment option. Responses were captured on a Microsoft Excell sheet and descriptively reported in percentages.

## 3 Results

100 questionnaires were analysed in this study with a 100% participation rate. The parental/guardian acceptance with regards to the use of silver fluoride is reported in Table 2. Majority of parents (74,5%) definitely agreed to the use of SF for their children. The use of SF on posterior teeth was more acceptable (74,5%) as opposed to its use on anterior teeth (43,1%). Parents agreed to the use of SF to reduce infection and pain (82,4%); avoid treatment under GA (84,3%); and to avoid an injection (72,5%). 52,9% of parents indicated their agreement in using SF not only because it has a reduced cost when compared to other treatments options but also because of its minimally invasive nature. The use of SF, even with Fluoride (82,4%) or Silver (80,4%) is almost totally accepted by parents and caregivers.

**Table 2:** Parental acceptance of SDF.

Variable	Definitely disagree	Neutral	Definitely agree
I would use silver diamine fluoride on my child.	9.8%	15.7%	74.5%
I would use silver diamine fluoride on my child to treat front teeth even if it turns teeth dark until it falls off.	31.3%	25.5%	43.1%
I would use silver diamine fluoride on my child to treat back teeth even if it turns teeth dark until it falls off.	17.6%	7.8%	74.5%
I would use silver diamine fluoride on my child if it can reduce the chances of infection and pain	-	13.7%	82.4%
I will use silver diamine fluoride on my child if it means avoiding treatment under general anesthesia	-	11.8%	84.3%
I would use silver diamine fluoride if my child can avoid having an injection	15.7%	11.8%	72.5%
I would use silver diamine fluoride if it reduces cost of treatment when compared to traditional fillings	17.6%	29.4%	52.9%
I would use silver diamine fluoride on my child even though it contains fluoride (within safety limits)	11.8%	-	82.4%
I would use silver diamine fluoride on my child even though it contains silver (within safety limits)	13.7%	-	80.4%

## 4 Discussion

SDF and SF are similar products with SF having some different clinical properties (12). Limited literature is available on SF as it is a recent newer generation. Parental acceptance of SF in this study, will therefore also be discussed with parental acceptance of SDF from previous literature.

Conventional dental procedures can often propagate fear due to the uncomfortable nature of some dental procedures and the ineffectiveness of certain behavioral management techniques among CSHCN. CSHCN suffering from caries can often only be treated under general anesthesia or sedation, thus predisposing them to multiple general anesthesia/sedation treatments in their lifetime (18, 19). Due to the medical conditions, sedation and general anaesthesia is often also considered high risk (19, 20). Brazilian parents indicated that they would consent to SF treatment if it will reduce infection/pain (82.4%), the need for injections (72.5%) and avoid the need for general anaesthesia (84.3%). Crystal et al. (21) and Hu et al. (17), reported similar results and demonstrated that parent's acceptance increases specifically to decrease their child's pain and suffering. Efforts should be made to optimize prevention and early management of carious lesions among CSHCN to limit pain and infection as well as the need for repeat sedation and general anaesthesia for dental treatments.

Authors corroborated that there is a higher acceptance of the use of SDF in primary teeth compared to permanent teeth, and posterior teeth compared to anterior teeth (22). Brazilian parents had similar views with only 43.1% definitely agreeing to the use of SF on anterior teeth but 74.5% on posterior teeth. The unaesthetic result (black colour) therefore remains a limitation of SDF/SF treatments on anterior teeth and should be discussed during patient education and obtaining consent.

Concerns with regards to the presence of fluoride and silver in SDF has been reported (23). The majority of Brazilian parents of CSHCN reported acceptance of the use of SF for caries management regardless of the product containing silver and fluoride. This is particularly relevant at this time when the safety of fluoride is under increased scrutiny, and prone to misinformation (24), which has the potential to reignite the debate of the use of fluoride to manage dental caries. It is important to note that there were however parents who were not comfortable with the use of silver (13.7%) and fluoride (11.8%), Efforts should be made to provide more awareness and education to parents on the constitution and safety of silver and fluoride in dental medicaments and should therefore also be included when obtaining informed consent for SDF/SF treatments.

Lastly, a limitation of the study is the small sample size and more research with a larger sample size is recommended to support the findings of this study. Secondly, the parental acceptance of SF was evaluated before the actual experience of the procedure. A previous study by Crystal et al. (21) has shown that when examining parental perception after SDF application, it was found that almost all of the respondents thought that SDF was acceptable when considering ease of application, providing more comfort for the child. Future studies could incorporate re-evaluation of the parents' acceptance after the child has undergone the SF treatment procedure.

## 5 Conclusion

Silver Fluoride was accepted by the majority of Brazilian parents of children with special health care needs. The positive response of parents and the minimally invasive nature of SF indicates that SF should be considered as a treatment option for caries limited to dentine for this vulnerable population. Patient education should include the unaesthetic result as well as product constitution to enable informed consent (25).

## Data Availability

The raw data supporting the conclusions of this article will be made available by the authors, without undue reservation.
